# Association between low-density cholesterol change and outcomes in acute ischemic stroke patients who underwent reperfusion therapy

**DOI:** 10.1186/s12883-021-02387-2

**Published:** 2021-09-16

**Authors:** Ting Cui, Changyi Wang, Qiange Zhu, Anmo Wang, Xuening Zhang, Shucheng Li, Yuan Yang, Wenzuo Shang, Rong Peng, Bo Wu

**Affiliations:** 1 Center of Cerebrovascular Diseases, Department of Neurology, West China Hospital, Sichuan University, No.37 Guo Xue Xiang, Sichuan 610041 Chengdu, China; 2 Departement of Rehabilitation Medicine Center, West China Hospital, Sichuan University, Chengdu, Sichuan China; 3 The Second Department of Neurology, Shanxi Provincial People’s Hospital, Xi’an, Shanxi China

**Keywords:** Low-density lipoprotein cholesterol, Change, Acute ischemic stroke, Reperfusion therapy, Outcome

## Abstract

**Background:**

Low-density lipoprotein cholesterol (LDL-C) can increase cardiovascular risk. However, the association between LDL-C change and functional outcomes in acute ischemic stroke (AIS) patients who underwent reperfusion therapy remains unclear.

**Methods:**

Patients who received reperfusion therapy were consecutively enrolled. LDL-C measurement was conducted at the emergency department immediately after admission and during hospitalization. The change of LDL-C level (ΔLDL-C) was calculated by subtracting the lowest LDL-C among all measurements during hospitalization from the admission LDL-C. Poor functional outcome was defined as modified Rankin Scale (mRS) > 2 at 90 days.

**Results:**

A total of 432 patients were enrolled (mean age 69.2 ± 13.5 years, 54.6 % males). The mean LDL-C level at admission was 2.55 ± 0.93 mmol/L. The median ΔLDL-C level was 0.43 mmol/L (IQR 0.08–0.94 mmol/L). A total of 263 (60.9 %) patients had poor functional outcomes at 90 days. There was no significant association between admission LDL-C level and functional outcome (OR 0.99, 95 % CI 0.77–1.27, *p* = 0.904). ΔLDL-C level was positively associated with poor functional outcome (OR 1.80, 95 % CI 1,12-2.91, *p* = 0.016). When patients were divided into tertiles according to ΔLDL-C, those in the upper tertile (T3, 0.80–3.98 mmol/L) were positively associated with poor functional outcomes compared to patients in the lower tertile (T1, -0.91-0.13 mmol/L) (OR 2.56, 95 % CI 1.22–5.36, *p* = 0.013). The risk of poor functional outcome increased significantly with ΔLDL-C tertile (*P*-trend = 0.010).

**Conclusions:**

In AIS patients who underwent reperfusion therapy, the decrease in LDL-C level during hospitalization was significantly associated with poor functional outcomes at 90 days.

**Supplementary Information:**

The online version contains supplementary material available at 10.1186/s12883-021-02387-2.

## Background

The association between low-density lipoprotein cholesterol (LDL-C) and outcomes in acute ischemic stroke (AIS) patients remains controversial [1–11]. The inconsistent results might be explained by oxidative stress. Since AIS patients may suffer from enhanced free-radical damage after reperfusion therapy [12, 13], focusing on patients with reperfusion therapy might clarify the role of LDL-C. However, previous studies on the association between LDL-C level and outcomes in AIS patients with reperfusion therapy failed to reach a consensus [5–8]. The conflicting conclusions may be due to the single measurement of LDL-C.

Some studies found that serum LDL-C levels decreased after the onset of AIS [14–18]. Under oxidative stress, low-density lipoprotein (LDL) gets oxidized into oxidized low-density lipoprotein (oxLDL) [19]. The extent of decreased LDL-C may reflect the degree of increased oxLDL, which may indicate the severity of oxidative stress [20] and is positively associated with poor functional outcomes [21–24]. Therefore, a more sensitive marker may be the change in serum LDL-C during hospitalization [25].

However, there is uncertainty on the association between LDL-C change and outcomes in patients with reperfusion therapy. In this study, we aimed to explore the association between changes in LDL-C levels and functional outcomes in these patients.

## Methods

### Study population

This is a retrospective study. AIS patients admitted to Neurology Department, West China Hospital were consecutively enrolled between 1st June 2018 and 31st January 2021. AIS was diagnosed based on clinical manifestation and brain image [26]. Patients were included as follows: (1) underwent reperfusion therapy within 6 h after symptom onset, including intravenous thrombolysis with alteplase and/or endovascular thrombectomy (including mechanical or thrombus aspiration thrombectomy, or both, with or without intra-arterial alteplase infusion), and (2) LDL-C levels were measured at emergency department immediately after admission and at least on another occasion during hospitalization. The exclusion criteria were as follows: (1) premorbid modified Rankin scale [mRS] scores > 1, (2) younger than 18 years, (3) had a liver injury that may affect serum lipid levels [15], or (4) malignancy. We obtained informed consent from each patient or their relative. The Scientific Research Department of West China Hospital approved this study.

### Baseline data

Data on demographics (age, gender), level of neurological severity (according to the National Institute of Health Stroke Scale [NIHSS] score), risk factors (atrial fibrillation, hypertension, hyperlipidemia, diabetes mellitus, smoking status, and coronary heart diseases), laboratory results (white blood cell, glucose, TG, TC, HDL, and LDL-C), and the interval between stroke onset and emergency department were documented at admission. The interval between stroke onset and admission measurement of LDL-C and the interval between admission and follow-up measurement of LDL-C during hospitalization were also documented. Serum LDL-C was measured by the automatic biochemistry analyzer (Roche Cobas 8000) [27]. The Trial of Org 10,172 in Acute Stroke Treatment (TOAST) classification system was conducted to identify stroke subtypes [28].

### Outcome

All patients were followed up by telephone or interview at 90 days to evaluate their functional outcomes blinded to their LDL-C levels. We used the modified Rankin Scale (mRS) to measure functional outcomes at 90 days [29]. Poor functional outcome was defined as mRS score > 2 [29].

### Statistical analysis

Continuous variables were reported as means with standard deviations (SD) for normally distributed parameters or medians with interquartile range (IQR) for non-normally distributed parameters. Frequencies or percentages were used to describe categorical variables. Descriptive analyses of study population baseline characteristics and 90-day outcomes were reported for groups using the χ2 test or Fisher’s exact test for categorical data, the Student’s t-test, and the Mann-Whitney U test for continuous variables as appropriate. Significant confounders were defined as variables within *p* < 0.10 in univariate analysis. The change of LDL-C level (ΔLDL-C) was calculated by subtracting the lowest LDL-C among all measurements during hospitalization from the admission LDL-C: a positive ΔLDL-C indicated LDL-C decreased during hospitalization, and a negative ΔLDL-C indicated an increase in LDL-C level. Multivariate logistic regression models were used to determine associations between ΔLDL-C and outcome. To further explore the associations, we did trend analyses by categorizing ΔLDL-C into tertiles [30]. Trends across tertiles (*P*-trend) of ΔLDL-C were determined by entering the median value of ΔLDL-C in each category as a continuous variable [31]. Data were reported as odds ratios (OR) and 95 % confidence intervals (CI). A two-sided *P* value less than 0.05 was considered statistically significant. All analyses were performed using IBM SPSS Statistics (25.0; IBM, Armonk, NY, USA).

## Results

### Baseline characteristics and outcome

**Fig. 1:**
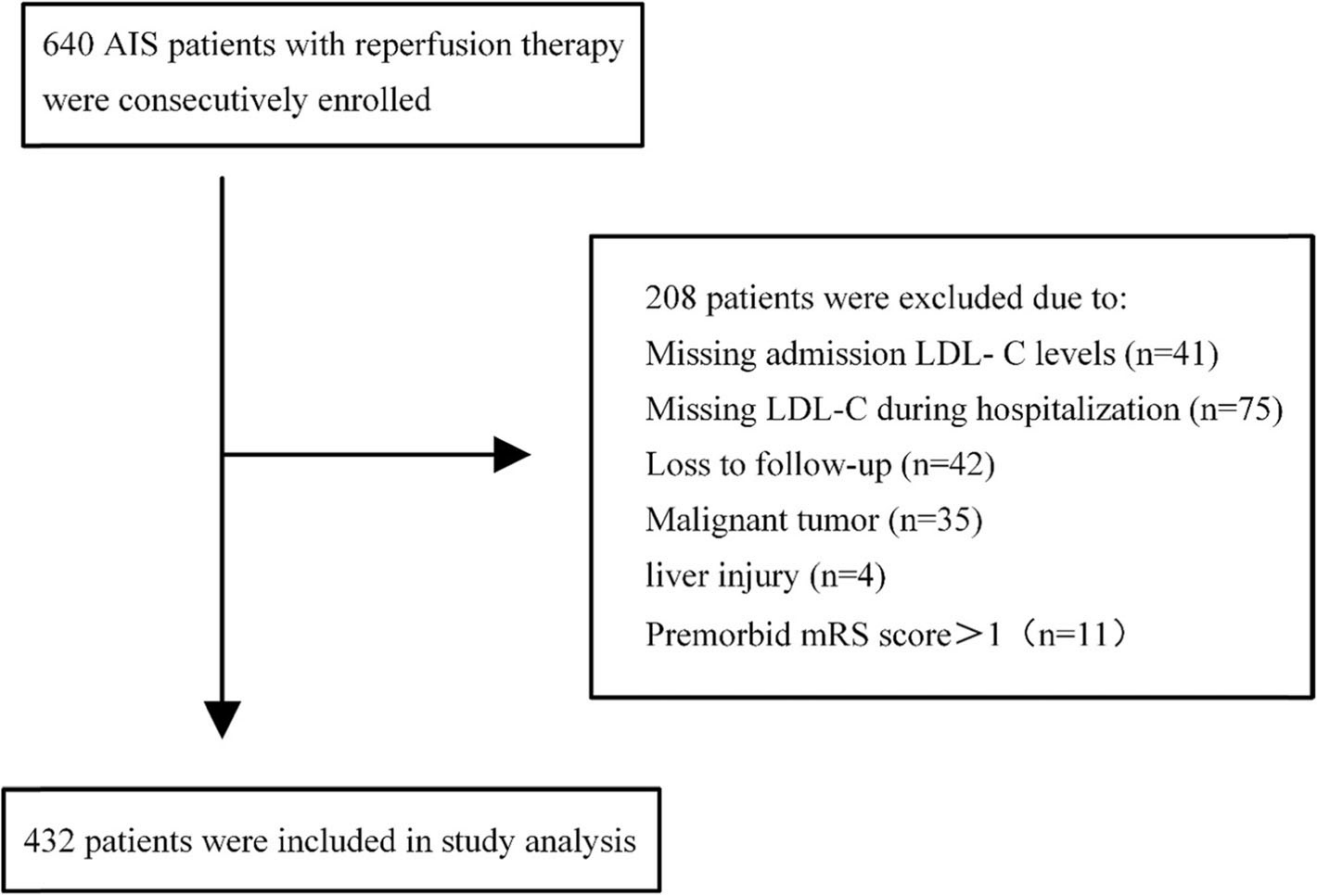
Patients’ inclusion flowchart. AIS, acute ischemic stroke; LDL-C, low-density lipoprotein cholesterol; mRS, modified Rankin Scale; ALT, alanine aminotransferase; AST, aspartate aminotransferase

As shown in Fig. 1, a total of 640 AIS patients underwent reperfusion therapy in our center, there were 24.6 % (158/640) patients missed LDL-C levels or outcome follow-up, we compared included patients to these patients, and we found that there were no significant differences in demographic parameters (age and gender), vascular risk factors (diabetes, atrial fibrillation, current smoking, and coronary heart diseases), baseline NIHSS score, TOAST classification, and reperfusion therapy method between two groups, except for more patients with prior history of stroke and hypertension among the included group (Table S1 in supplementary materials). Finally, a total of 432 patients (mean age 69.2 ± 13.5 years, 54.6 % males) were included. As shown in Table 1, the mean admission LDL-C level was 2.55 ± 0.93 mmol/L, the mean lowest LDL-C level during hospitalization was 2.00 ± 0.88mmol/L, and the median ΔLDL-C was 0.43 mmol/L (IQR 0.08–0.94 mmol/L). The median interval time between stroke onset and emergency department was 2.5 h (IQR 1.8-3.0 h). The median interval time between admission and the lowest LDL-C measurement during hospitalization was 3.1 d (IQR 0.8–6.6 d). For most patients (357/432, 82.6 %), LDL-C levels decreased during hospitalization. A total of 263 (60.9 %) patients had poor 90-day functional outcomes.

**Table 1:** Patient characteristics stratified by functional outcome at 90 days

Variables	Overall	Good functional outcome	Poor functional outcome	*P* value
	(*n* = 432)	(*n* = 169)	(*n* = 263)	
Age, years, mean (SD)	69.2 (13.5)	64.9 (13.9)	72.0 (12.5)	< 0.001
Male, n (%)	236 (54.6)	109 (64.4)	127 (48.3)	< 0.001
Hypertension, n (%)	259 (60.0)	95 (56.2)	164 (62.4)	0.203
Diabetes, n (%)	99 (22.9)	34 (20.1)	65 (24.7)	0.267
hyperlipemia, n (%)	34 (7.9)	13 (7.7)	21 (8.0)	0.912
Atrial fibrillation, n (%)	216 (50.0)	63 (37.3)	133 (58.2)	< 0.001
Valvular heart diseases, n (%)	74 (17.1)	25 (14.8)	49 (18.6)	0.301
Coronary heart diseases, n (%)	63 (14.6)	22 (13.0)	41 (15.6)	0.460
Previous stroke, n (%)	39 (9.0)	12(7.1)	27 (10.3)	0.263
Current smoking, n (%)	107 (24.8)	52 (30.8)	55 (20.9)	0.021
Alcohol consumption, n (%)	100 (23.1)	52 (30.8)	51 (19.4)	0.021
Statin use before admission, n (%)	39 (9.0)	14 (8.3)	25 (9.5)	0.665
Baseline NIHSS, median (Q1-Q3)	14 (9–18)	9 (6–14)	15(12–20)	< 0.001
LDL-C, mmol/L, mean (SD)	2.55 (0.93)	2.59 (0.91)	2.53 (0.95)	0.496
HDL, mmol/L, mean (SD)	1.27 (0.42)	1.25 (0. 49)	1.29 (0.36)	0.332
TG, mmol/L, median (Q1-Q3)	1.24 (0.87–1.89)	1.20 (0.85–1.94)	1.20 (0.88–1.81)	0.566
TC, mmol/L, mean (SD)	4.23 (1.13)	4.22 (1.13)	4.23 (1.13)	0.966
Serum glucose, mmol/L, mean (SD)	7.53 (6.49–9.18)	7.96 (2.76)	8.57 (3.04)	0.035
White blood cell, ^a^10^9 /L, mean (SD)	8.47 (3.19)	8.23 (2.77)	8.62 (3.43)	0.207
TOAST classification, n (%)				< 0.001
Large-artery Atherosclerosis	139 (32.2)	59 (34.9)	80 (30.4)	
Cardio-embolism	192(44.4)	57 (33.7)	135 (51.3)	
Lacunar	28 (6.5)	22 (13.0)	6 (2.3)	
Other	13 (3.0)	7 (4.1)	6 (2.3)	
Undetermined	60 (13.9)	24 (14.2)	36 (13.7)	
Reperfusion therapy method, n (%)				0.092
thrombolysis only	124 (28.7)	58 (34.3)	66 (25.1)	
thrombectomy only	210 (48.6)	70 (41.4)	140 (53.2)	
thrombolysis and thrombectomy	*9*8 (22.7)	41 (24.3)	57 (21.7)	
Statin use during hospitalization, n (%)	263 (60.9)	103 (60.9)	160 (60.8)	0.982
Interval between stroke onset and emergency department, h, median (Q1-Q3)	2.5 (1.8-3.0)	2.3 (2.0–3.0)	2.5 (1.7–3.5)	0.610
Interval between stroke onset and admission measurement of LDL-C, h, median (Q1-Q3)	3.1 (2.2–3.9)	3.1 (2.1–3.8)	3.1 (2.1–3.8)	0.685
Interval between admission and follow-up measurement of LDL-C, d, median (Q1-Q3)	3.1 (0.8–6.6)	1.7 (0.6–6.4)	3.4 (1.0-7.1)	0.008
^a^ΔLDL-C, mmol/L, median (Q1-Q3)	0.43 (0.08–0.94)	0.28 (0.07–0.78)	0.52 (0.10–1.03)	0.009
The lowest LDL-C during hospitalization, mmol/l, median (Q1-Q3)	1.92 (1.38–2.44)	2.11 (1.48–2.69)	1.83 (1.34–2.32)	0.002
LDL-C variation, n (%)				0.542
decreased (ΔLDL-C > 0)	357 (82.6)	142 (84.0)	215 (81.7)	
increased (ΔLDL-C ≤ 0)	75 (17.4)	27 (16.0)	48 (18.3)	

*SD* standard deviation, *LDL-C* low-density lipoprotein cholesterol, *ΔLDL-C* the change of low-density lipoprotein cholesterol during hospitalization, *NIHSS* National Institutes of Health Stroke Scale, *TOAST* the Trial of Org 10,172 in Acute Stroke Treatment

^a^ΔLDL-C was calculated by subtracting the lowest LDL-C among all measurements during hospitalization from the admission LDL-C

### Association between admission LDL-C and outcome

Age, sex, baseline NIHSS score, atrial fibrillation, current smoking, drinking consumption, TOAST classification, serum glucose, reperfusion therapy method, and the interval between admission LDL-C and the lowest LDL-C during hospitalization were significantly correlated with poor outcomes in univariate analysis (Table 2). There was no significant association between admission LDL-C level and functional outcome at 90 days when LDL-C level was regarded as a continuous variable (OR 1.03, 95 % CI 0.81–1.31, *p* = 0.802), or categorical variable (T3 vs. T1, OR 0.97, 95 % CI 0.55–1.71, *p* = 0.919, Table 3).

**Table 2:** Univariable logistic regression analysis of variables associated with poor functional outcome

Variable	Unadjusted odds ratio (95% confidence interval)	*p*-value
Age	1.04 (1.03, 1.06)	<0.001
Male	0.51 (0.35, 0.77)	0.001
Hypertension	1.29 (0.87, 1.91)	0.204
Diabetes	1.30 (0.82, 2.08)	0.268
hyperlipemia	1.04 (0.51, 2.14)	0.912
Atrial fibrillation	2.34 (1.57, 3.48)	<0.001
Valvular heart diseases	1.32 (0.78, 2.23)	0.302
coronary heart diseases	1.23 (0.71, 2.16)	0.460
Statin use before admission	1.16 (0.59, 2.31)	0.666
Previous stroke	1.50 (0.74, 3.04)	0.265
Current smoking	0.60 (0.38, 0.93)	0.021
Alcohol consumption	0.59 (0.38, 0.93)	0.022
Baseline NIHSS score	1.17 (1.12, 1.21)	<0.001
HDL upon admission	1.28 (0.78, 2.10)	0.334
TG upon admission	0.92 (0.78, 1.09)	0.333
TC upon admission	1.00 (0.85, 1.19)	0.966
Serum glucose	1.08 (1.00, 1.17)	0.038
White blood cell	1.04 (0.98, 1.11)	0.208
TOAST classification		
Large-artery Atherosclerosis	Reference	
Cardio-embolism	1.75 (1.11, 2.76)	0.017
Lacunar	0.20 (0.08, 0.53)	0.001
Other	0.63 (0.20, 1.98)	0.431
Undetermined	1.11 (0.60, 2.05)	0.748
Reperfusion therapy method		
thrombolysis only	Reference	
thrombectomy only	1.76 (1.12, 2.77)	0.015
thrombolysis and thrombectomy	1.22 (0.72, 2.09)	0.463
Statin use during hospitalization	0.98 (0.67, 1.48)	0.995
Interval between stroke onset and emergency department	1.05 (0.89, 1.24)	0.546
Interval between stroke onset and admission measurement of LDL-C	1.04 (0.89, 1.21)	0.616
Interval between admission and follow-up measurement of LDL-C	1.06 (1.01, 1.12)	0.023

*NIHSS* National Institutes of Health Stroke Scale, *TOAST* the Trial of Org 10,172 in Acute Stroke Treatment

**Table 3:** Multivariate logistic regression analysis between admission LDL-C and poor functional outcome^a^

Variable	Non-adjusted model	Adjusted model
Admission LDL-C, mmol/L	0.93 (0.76, 1.14), 0.496	1.03 (0.81, 1.31), 0.802
Admission LDL-C tertiles, mmol/L		
T1(0.74–2.15)	Reference	Reference
T2(2.16–2.80)	1.31 (0.81, 2.12), 0.271	1.62 (0.92, 2.85), 0.096
T3(2.81–9.61)	0.82 (0.51, 1.31), 0.404	0.97 (0.55, 1.71), 0.919

Adjusted model: adjusted for age, sex, atrial fibrillation, Current smoking, drinking consumption, baseline NIHSS score, Serum glucose, TOAST classification, and reperfusion therapy method

*LDL-C* low-Density Lipoprotein Cholesterol, *NIHSS* National Institutes of Health Stroke Scale, *TOAST* the Trial of Org 10,172 in Acute Stroke Treatment

^a^Results for each model are presented as odds ratio (95 % confidence interval), *p*-value

### Association between ΔLDL-C and outcome

When ΔLDL-C was regarded as a continuous variable, ΔLDL-C was significantly associated with poor functional outcome at 90 days in univariate analysis (OR 1.55, 95 % CI 1.12–2.15, *p* = 0.009, Table 4). After adjusting for confounding variables, the association between ΔLDL-C and the poor outcome remained significant (OR 1.80, 95 % CI 1.12–2.91, *p* = 0.016).

When ΔLDL-C was regarded as a categorical variable, patients in the upper tertile (T3, 0.80–3.98 mmol/L) had a higher risk of poor outcome than those in the lower tertile (T1, -0.91-0.13 mmol/L) in univariate analysis (OR 1.92, 95 % CI 1.15–3.20, *p* = 0.012). After adjusting for confounding variables, the association between ΔLDL-C and the poor outcome remained significant (OR 2.56, 95 % CI 1.22–5.36, *p* = 0.013). The risk of poor functional outcome increased significantly with ΔLDL-C tertile (*P*-trend = 0.010).

**Table 4:** Multivariate logistic regression analysis between ΔLDL-C and poor functional outcome^a^

Variable	Non-adjusted model	Adjusted model 1	Adjusted model 2
ΔLDL-C, mmol/l	1.55(1.12, 2.15),0.009	1.79(1.11, 2.89),0.017	1.80(1.12, 2.91),0.016
ΔLDL-C tertiles, mmol/l			
T1 (-0.91-0.13)	Reference	Reference	Reference
T2 (0.14–0.79)	1.03(0.65, 1.64),0.888	1.23(0.70, 2.18),0.473	1.24(0.70, 2.19),0.470
T3 (0.80–3.98)	1.92(1.15, 3.20),0.012	2.56(1.22, 5.35),0.013	2.56(1.22, 5.36),0.013
*P*-trend	0.007	0.010	0.010

Adjusted model 1: adjusted for age, sex, atrial fibrillation, Current smoking, drinking consumption, baseline NIHSS score, Serum glucose, TOAST classification, reperfusion therapy method, and interval between admission and follow-up measurement of LDL-C

Adjusted model 2: adjusted for variables in model 1 and statin use during hospitalization

*LDL-C* low-density lipoprotein cholesterol, *ΔLDL-C* the change of low-density lipoprotein cholesterol during hospitalization, *NIHSS* National Institutes of Health Stroke Scale, *TOAST* the Trial of Org 10,172 in Acute Stroke Treatment

^a^Results for each model are presented as odds ratio (95 % confidence interval), *p*-value

## Discussion

We found that the admission LDL-C was not associated with functional outcomes at 90 days. For most AIS patients who underwent reperfusion therapy, LDL-C decreased during hospitalization. The decrease of LDL-C during hospitalization was associated with poor 90-day functional outcomes. We suggested that the magnitude of decrease in LDL-C during hospitalization may reflect the severity of oxidative stress in the acute phase of AIS generated by ischemic stroke and/or brain tissue reperfusion, which might be positively associated with poor 90-day functional outcome in AIS patients with reperfusion therapy.

There was a discrepancy in the prognostic significance between LDL-C level and outcomes in AIS patients [1–11]. Some studies found that higher LDL-C level was associated with poor outcomes in AIS patients [1–3], while some studies found that lower LDL-C was associated with poor outcome in AIS patients [4, 5], others failed to find a significant association between LDL-C and outcome [6–11]. The inconsistent results might be explained by differences in sample size, patient selection, potential confounder, outcome assessment, and different measurement times of LDL-C. Several studies suggested that LDL got oxidized into oxLDL under oxidative stress [19], and oxLDL may contribute to exacerbate free-radical damage in the acute phase of AIS [20, 32–35]. Since AIS patients with reperfusion therapy could suffer from enhanced oxidative injury [12], focusing on these patients might clarify the role of LDL-C.

However, previous studies on the association between LDL-C level and outcomes in AIS patients who underwent reperfusion therapy were rare, and these conclusions failed to reach a consensus [5–8]. Previous studies of AIS patients with thrombolytic therapy failed to find an association between baseline LDL-C and outcome [6–8], which was in line with our study. Recently, a retrospective study involving 174 AIS patients with endovascular thrombectomy (EVT) therapy found that a higher LDL-C level at admission was independently associated with favorable functional outcomes at 3 months [5]. The conflicting results in AIS patients with reperfusion therapy may be due to the single measurement of LDL-C.

Previous studies suggested that LDL-C levels showed a decreased trend during the acute phase of AIS [14–18]. However, only one study investigated the association between LDL-C change and outcomes in AIS patients [25]. This multicenter study of 676 AIS patients found that increased LDL-C was associated with poor outcomes at discharge [25]. For most patients (566/676, 83.7 %), LDL-C levels decreased during hospitalization in this study, but it did not clarify the association between decreased LDL-C and outcome further. In the current study, we also found that for most patients (357/432, 82.6 %), LDL-C levels decreased during hospitalization, and decreased LDL-C level was significantly associated with poor 90-day functional outcome in AIS patients with reperfusion therapy.

Though the underlying mechanism of the association between ΔLDL-C and outcome remains unclear, it could be explained as follows: LDL-C gets oxidized into oxLDL under oxidative stress [19] and oxLDL is the major marker of oxidative stress [20, 32–34]. Previous studies found that high oxLDL is positively associated with poor functional outcomes in AIS patients [21–23]. Therefore, we speculated that the increased oxLDL level may be associated with decreased LDL-C level during hospitalization, the extent of decreased LDL-C may reflect the degree of increased oxLDL, which may indicate the severity of oxidative stress and contribute to poor functional outcomes. Although the specific mechanism of ΔLDL-C level during hospitalization remains unclear, during the oxidative challenge, LDL-C gets oxidized into oxLDL [19], which contributes to free-radical damage [20, 32–35] and poor outcome [21–23].

A study of 3019 AIS /TIA patients from the Clopidogrel in High-Risk Patients with Acute Non-Disabling Cerebrovascular Events (CHANCE) trial found that higher levels of ox-LDL and ox-LDL/LDL significantly increased the risk of poor functional outcome in AIS patients [21], which may provide evidence to support our hypothesis. Of course, more studies of high quality are needed to verify the above hypothesis.

From a clinical point of view, since LDL-C is a widely available biomarker and is measured frequently, our findings might help clinicians to identify AIS patients who underwent reperfusion therapy at risk of 90-day poor functional outcome and guide therapy properly, besides there was no additional financial burden for patients’ families.

Some limitations should be noted. Firstly, this was a retrospective study and we could not measure oxLDL. Therefore, we could not confirm that the change in LDL-C levels was due to free radical damage; however, a previous study found that the higher levels of oxLDL, and ox-LDL/LDL-C significantly increased the risk of poor outcome [21], which supported our hypothesis. Secondly, we could not measure LDL-C at a specific time for each patient. The results might vary with different testing times [16]. However, in multivariate analysis, we adjusted the interval between admission and follow-up measurement of LDL-C during hospitalization in Model1, and our results remain significant in the present study. Thirdly, statin therapy could have influenced LDL-C levels. A randomized controlled trial of 60 AIS patients found that LDL-C decreased significantly in statin-treated patients on the 7th day and 3 months [17]. In our study, the median interval between admission and the lowest LDL-C level measurement was 3.1d (IQR 0.8–6.6 d). In addition, there was no significant association between statin usage and outcome in univariate analysis. Moreover, when we further adjusted for this variable in Model 2, our findings remained significant. Therefore, the influence of statin usage may be limited in our study. Fourthly, we measured LDL-C in a non-fasting state, which might influence the results, but a meta-analysis of 68 studies found that the association between LDL-C and ischemic stroke remained even when measured in non-fasting patients [36]. Finally, patients did not conduct a computed tomographic angiography after reperfusion therapy in our hospital, therefore we could not evaluate the status of their blood vessels, which might influence our results.

## Conclusions

There was no significant association between admission LDL-C level and outcomes in AIS patients who underwent reperfusion therapy, while the decrease in LDL-C level during hospitalization was positively associated with poor functional outcomes at 90 days.

## Supplementary Information

**Additional file 1: Table S1.** Patient characteristics stratified by included patients and excluded patients

## Data Availability

The data that support the findings of this study are available from the corresponding author upon reasonable request.

## Abbreviations

AIS: Acute ischaemic stroke
LDL-C: Low-density lipoprotein cholesterol
ΔLDL-C: The variation of Low-density lipoprotein cholesterol during hospitalization
NIHSS: National Institutes of Health Stroke Scale
OR: Odds ratio
CI: Confidence interval
HDL: High-density lipoprotein cholesterol
TC: Total cholesterol
TG: Triglycerides
TOAST: The Trial of ORG 10172 in Acute StrokeTreatment
