# Supplementary material for: Association between low-density cholesterol change and outcomes in acute ischemic stroke patients who underwent reperfusion therapy
Source: BMC Neurol. 2021 Sep 16;21:360. doi: 10.1186/s12883-021-02387-2 (PMC8447794; doi:10.1186/s12883-021-02387-2)
Supplement: Supplementary file 1 — Additional file 1: Table S1. Patient characteristics stratified by included patients and excluded patients [file 12883_2021_2387_MOESM1_ESM.docx]

Table S1. Patient characteristics stratified by included patients and excluded patients

|  | Overall  (n=590) | excluded*  (n=158) | included  (n=432) | P value |
| --- | --- | --- | --- | --- |
| Male, n (%)  Age, years, mean (SD) | 321 (54.4)  68.6 (13.9) | 85 (53.8)  66.9 (14.9) | 236 (54.6)  69.2 (13.5) | 0.931  0.076 |
| Interval between stroke onset and emergency department, h, median (Q1-Q3) | 2.5 (1.5-3.4) | 2.3 (0.9-3.7) | 2.5 (1.8-3.0) | 0.258 |
| Baseline NIHSS, median (Q1-Q3) | 14 (8-18) | 13 (8-18) | 14 (9-18) | 0.172 |
| Reperfusion therapy method, n (%) | |  |  | 0.064 |
| thrombolysis only | 174 (29.5) | 50 (31.6) | 124 (28.7) |  |
| thrombectomy only | 296 (50.2) | 86 (54.4) | 210 (48.6) |  |
| thrombolysis and  thrombectomy | 120 (20.3) | 22 (13.9) | 98 (22.7) |  |
| Statin use before admission, n (%) | 49 (8.3) | 10 (6.3) | 39 (9.0) | 0.377 |
| Current smoking, n (%) | 149 (25.3) | 42 (26.6) | 107 (24.8) | 0.732 |
| Alcohol consumption, n (%) | 140 (23.7) | 40 (25.3) | 100 (23.1) | 0.661 |
| Hypertension, n (%) | 338 (57.3) | 79 (50.0) | 259 (60.0) | 0.038 |
| Diabetes, n (%) | 136 (23.1) | 37 (23.4) | 99 (22.9) | 0.986 |
| Atrial fibrillation, n (%) | 283 (48.0) | 67 (42.4) | 216 (50.0) | 0.123 |
| Previous stroke, n (%) | 43 (7.3) | 4 (2.5) | 39 (9.0) | 0.012 |
| hyperlipemia, n (%) | 47 (8.0) | 13 (8.2) | 34 (7.9) | 1.000 |
| Coronary heart diseases, n (%) | 83 (14.1) | 20 (12.7) | 63 (14.6) | 0.644 |
| Valvular heart diseases, n (%) | 107 (18.1) | 33 (20.9) | 74 (17.1) | 0.353 |
| TOAST classification, n (%) |  |  |  | 0.623 |
| Large-artery Atherosclerosis | 191 (32.4) | 52 (32.9) | 139 (32.2) |  |
| Cardio-embolism | 257 (43.6) | 65 (41.1) | 192 (44.4) |  |
| Lacunar | 39 (6.6) | 11 (7.0) | 28 (6.5) |  |
| Other | 22 (3.7) | 9 (5.7) | 13 (3.0) |  |
| Undetermined | 81 (13.7) | 21 (13.3) | 60 (13.9) |  |

SD, standard deviation; NIHSS, National Institutes of Health Stroke Scale; TOAST: the Trial of Org 10172 in Acute Stroke Treatment.

* Only patients excluded by missing LDL-C levels or outcome follow-up were analyzed.
